# Utility of Restricted Mean Survival Time for Analyzing Time to Nursing Home Placement Among Patients With Dementia

**DOI:** 10.1001/jamanetworkopen.2020.34745

**Published:** 2021-01-28

**Authors:** Dae Hyun Kim, Xihao Li, Shijia Bian, Lee-Jen Wei, Ryan Sun

**Affiliations:** 1 Hinda and Arthur Marcus Institute for Aging Research, Hebrew SeniorLife, Boston, Massachusetts; 2 Division of Gerontology, Beth Israel Deaconess Medical Center, Boston, Massachusetts; 3 Division of Pharmacoepidemiology and Pharmacoeconomics, Brigham and Women’s Hospital, Boston, Massachusetts; 4 Department of Biostatistics, Harvard T.H. Chan School of Public Health, Boston, Massachusetts; 5 Department of Biostatistics and Bioinformatics, Rollins School of Public Health, Emory University, Atlanta, Georgia; 6 Department of Biostatistics, The University of Texas MD Anderson Cancer Center, Houston

## Abstract

This cohort study assesses the utility of restricted mean survival time as a method for quantifying time to nursing home placement among patients with dementia.

## Introduction

Delaying nursing home (NH) placement for patients with dementia is an important goal. The effect of a treatment for dementia on NH placement is conventionally summarized as a hazard ratio, risk difference, or median time difference. Recently, restricted mean survival time (RMST) has been proposed as an intuitive measure that summarizes treatment effect in terms of the difference in the number of event-free days.^1,2,3^ Despite its advantages over conventional measures (Table), to date, RMST has not been applied to clinical trials of dementia treatments. We assessed the utility of RMST in analyzing time to NH placement among patients with dementia using the Donepezil and Memantine in Moderate to Severe Alzheimer’s Disease (DOMINO-AD) trial as an example.^4,5^

**Table.** Measures of Treatment Effect for Nursing Home Placement in Clinical Trials of Treatments for Dementia^a^

Measure	Strengths and limitations	DOMINO-AD result, estimate (95% CI)^b^
Hazard ratio	Hazard rate and ratio of 2 hazard rates (without a reference hazard rate) are not intuitive to interpret, proportional hazards are assumed (treatment effect is constant over time), and statistical power depends on the number of events	1.29 (0.95 to 1.75)
Risk difference, %	Risk difference is intuitive to interpret, and risk at a fixed time point (eg, end of study) may not capture a short-term yet meaningful delay in events	0.2 (−12.5 to 13.2)^c^
Median time difference, d	Median event-free time is intuitive to interpret, is a “local” summary (50th percentile of the distribution) that is insensitive to outliers (early or late events), and is often less precise (ie, wide CI) and not estimable when the event rate is low (<50%)	−135 (−385 to 63)
RMST, d	A gain or loss in the event-free time within a fixed period (eg, study duration) is intuitive to interpret, RMST corresponds to the area under the Kaplan-Meier curve, the time window needs to be prespecified, statistical power depends on the exposed follow-up time, and RMST can provide a more precise estimate (ie, narrow CI) in case of a low event rate	−108 (−240 to 23)

Abbreviations: DOMINO-AD, Donepezil and Memantine in Moderate to Severe Alzheimer’s Disease; RMST, restrictive mean survival time.

^a^ Treatment effect estimates were calculated from reconstructed DOMINO-AD trial data (see the Methods section for details).

^b^ Donepezil discontinuation vs continuation.

^c^ For treatment discontinuation vs continuation, the risk difference was 36.8% vs 21.4% at 1 year and 76.7% vs 76.5% at 4 years.

## Methods

This cohort study consisted of a post hoc analysis aimed to assess use of RMST methods; thus, we did not obtain data from the DOMINO-AD investigators but reconstructed data by scanning Kaplan-Meier curves from the study by Howard et al^5^ using WebPlotDigitizer software (https://automeris.io/WebPlotDigitizer) and applying a validated algorithm.^6^ This algorithm reconstructs patient-level data based on the number of patients at risk and the magnitudes and locations of steps in the Kapan-Meier curves. The Hebrew SeniorLife/Advarra Institutional Review Board deemed that institutional review board review was not necessary because the study used reconstructed data. This study followed the Strengthening the Reporting of Observational Studies in Epidemiology (STROBE) reporting guideline.

The DOMINO-AD trial enrolled 295 patients with moderate to severe Alzheimer disease in England and Scotland. The mean (SD) age of the patients was 77.1 (8.4) years; 35% of patients were male, and 95% were White. All patients had received donepezil for at least 3 months and were randomly assigned to 1 of 4 groups: (1) donepezil and memantine (n = 73), (2) donepezil without memantine (n = 73), (3) memantine without donepezil (n = 76), or (4) discontinuation of both drugs (n = 73). Nursing home placement was a secondary outcome. Because the rate of NH placement did not differ according to treatment with memantine, we estimated hazard ratio, risk difference, median event-free time difference, and RMST difference for NH placement over 4 years (or 1460 days) and their 95% CIs between donepezil continuation and discontinuation arms. Analyses were performed using R, version 4.0.0 (R Project for Statistical Computing) and packages reconstructKM and surv2sampleComp.

## Results

Over 4 years, 162 participants were placed in an NH. The Kaplan-Meier curves show that the risk of NH placement among those who discontinued donepezil treatment was initially higher (36.8% vs 21.4% at 1 year) but later became similar to that for those who continued the treatment (76.7% vs 76.5% at 4 years), suggesting a withdrawal effect (Figure). This delay in NH placement could not be inferred or quantified from the hazard ratio (discontinuation vs continuation: 1.29; 95% CI, 0.95-1.75) or risk difference (0.2%; 95% CI, −12.5% to 13.2%) (Table). Differences in the median NH-free time (−135 days; 95% CI, −385 to 63 days) and RMST (mean NH-free time, −108 days; 95% CI, −240 to 23 days) suggested a delay in NH placement.

**Figure.**
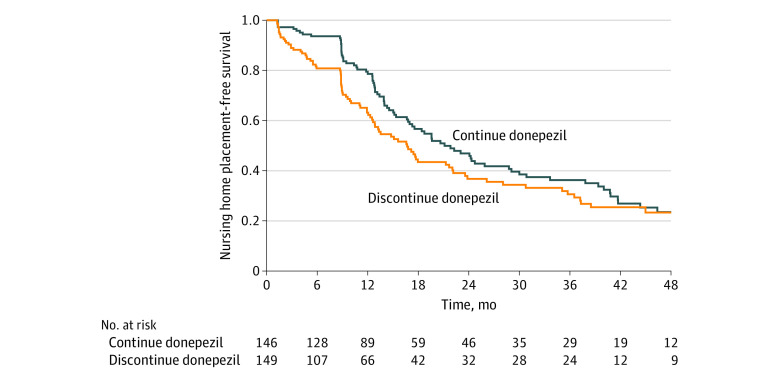
Continuation and Discontinuation of Donepezil and Nursing Home (NH) Placement–Free Survival in the Donepezil and Memantine in Moderate to Severe Alzheimer’s Disease Trial The area under the survival curve from baseline to 48 months was 800 days for continuation of donepezil and 692 days for discontinuation of donepezil, with a difference of 108 days.

## Discussion

In this cohort study, unlike hazard ratios and risk differences, RMST quantified a benefit of donepezil continuation in terms of an associated gain or loss in NH-free days. The median NH-free time difference, although intuitive, was insensitive to early or late NH placement and had a wider 95% CI than that did RMST. The 95% CI of the RMST difference did not rule out the possibility of a clinically meaningful delay (up to 237 days) in NH placement. Although RMST analysis assumes noninformative censoring and requires a priori determination of a time window, it does not require the proportional hazards assumption of a Cox proportional hazards regression model, which was violated in the DOMINO-AD trial.^5^ Because our objective was to assess use of RMST methods, one limitation of our analysis was that it was not intended to comprehensively evaluate benefits vs harms (eg, need for a pacemaker) associated with donepezil or to offer new clinical insights. Nonetheless, for patients with Alzheimer disease, the majority of whom may ultimately require NH placement, our findings suggest that RMST may complement conventional measures of treatment effect by helping to estimate a potential delay in NH placement.
