# Error in the Discussion Section

**DOI:** 10.1001/jamanetworkopen.2021.3081

**Published:** 2021-03-02

In the Research Letter titled “Utility of Restricted Mean Survival Time for Analyzing Time to Nursing Home Placement Among Patients With Dementia,”^1^ published January 28, 2021, in the Discussion section, the time for a clinically meaningful delay in nursing home placement was changed from up to 237 days to up to 240 days. This article has been corrected.
